# Status analysis of quality control of administered infusion solution with cytotoxic drugs

**DOI:** 10.3389/or.2024.1415677

**Published:** 2025-01-06

**Authors:** Shan Wang, Feng-Ying Zhang, Xue Dou, Xiao-Lin Pan, Chen Su, Jin-Lei Tian, Dian-Ping Mu

**Affiliations:** ^1^ Department of Pharmacy, Tianjin First Central Hospital, Nankai University, Tianjin, China; ^2^ College of Chemistry, Nankai University, Tianjin, China

**Keywords:** administered infusion solution, cytotoxic drugs, physical stability, chemical stability, sterility

## Abstract

The administered infusion solution is a sterile preparation that can be used directly for intravenous infusion in patients by mixing one or more intravenous drugs using aseptic operation technology. The pharmacy intravenous admixture service (PIVAS) center is a professional technical service department in hospitals, where the majority of inpatient-administered infusion solutions are prepared. During the processes of dissolution, dilution, preparation, storage, and use of intravenous drugs, the quality control of the administered infusion solution can be affected by various factors. At present, there are no relevant standards or guidance documents for the quality control of administered infusion solutions. Cytotoxic drugs are still the main treatment option for cancer patients and are mainly prepared in PIVAS centers in most hospitals. In this study, we mainly focused on the quality control of cytotoxic drug-administered infusion solutions and explored associated factors (diluent, container, concentration, temperature, and light), physical stability (visual appearance, pH, osmolality, and particulate matter), chemical stability (content), and biological stability (sterility). Most of the studies reviewed in this paper have insufficient data on the related factors and physicochemical stability of the administered infusion solutions. Research on the sterility of administered infusion solutions is particularly limited, with only one article addressing this aspect. Ensuring the quality of cytotoxic drug-administered infusion solutions is vital for the safe administration of drugs to cancer patients, so it is very important to enhance associated research. This article summarized the relevant literature on the quality control of cytotoxic drug-administered infusion solutions and provided a reference for safer and more efficient use of these drugs in clinical practice.

## 1 Introduction

Intravenous infusion is one of the most important treatments and is commonly used in clinical practice, particularly for hospitalized patients. In 2020, the intravenous infusion rate of inpatients in secondary and higher-level hospitals was 86.10% in China (1). The utilization rate of venous infusion in inpatients is relatively high. According to the Annual Report of National Adverse Drug Reaction Monitoring (2022) in China, adverse drug reactions (ADRs) related to intravenous administration accounted for 57.8% (2). Oral administration exhibits highly variable pharmacokinetics. In order to increase bioavailability, intravenous administration is required in clinical practice. The pharmacy intravenous admixture service (PIVAS) center is a professional technical service department in hospitals that provides high-quality intravenous infusion to ensure safety and effectiveness. PIVAS centers are equipped with clean dispensing environments, advanced dispensing technologies, and professional medication order review systems, where pharmacists participate in the whole process. The PIVAS workflow is complex and mainly involves the following steps: the clinician prescribes intravenous therapy→ order information transfer→ receipt of PIVAS workstation→ the pharmacist reviews the order→ print label→ dispensing→ dispensing check→ mixing→ administered infusion solution check→ administered infusion solution packaging→ place in closed containers and lock→ sent to the ward. In order to avoid medication errors and decrease ADRs related to infusion, the PIVAS center incorporates two important steps: dispensing check and administered infusion solution check.

An administered infusion solution is a mixture of one or more types of intravenous drugs prepared using aseptic operation technology (to prevent bacteria from entering the human body or other objects), which can be directly used for intravenous infusion in patients in clinical settings. The administered infusion solution check is the last step in the PIVAS workflow, where the solution is verified according to specific standards for different drugs. This check includes examining the appearance of the infusion bag or bottle for cleanliness or leaks; assessing whether the administered infusion solution shows signs of discoloration, turbidity, precipitation, or crystallization; confirming whether the basic infusion and empty bottle match the drug name, specification, and dosage indicated on the label; and checking the rationality of drug compatibility and the suitability of the drug dosage. The visual appearance of the administered infusion solution is mainly considered. However, the time and conditions of storage will directly affect the quality of the administered infusion solution. In practice, more detailed information is often required about the administered infusion solution; for example, fluorouracil may need to be administered for several days to maintain steady plasma concentrations (3). Furthermore, some drugs such as monoclonal antibodies are very expensive, and in some special cases, their administered infusion solution cannot be used in a timely manner. However, the lack of stability data leads to drug waste. Thus, there is a compelling need for more comprehensive data on the practical use of administered infusion solutions (4).

Cancer remains a major public health concern in China, according to the latest report by the National Cancer Center (NCC) of China (5). Although targeted and monoclonal antibody drugs have been used in the treatment of tumors in recent years, cytotoxic drug-mediated chemotherapy is still the mainstream approach for tumor treatment. Cytotoxic agents not only target tumor cells but also damage healthy cells; for pharmacists and nurses, long-term exposure to these cytotoxic agents will cause certain damage to the normal body (6). To avoid occupational exposure to cytotoxic drugs, their reconstitution and preparation take place in PIVAS centers under strict aseptic conditions and were finally sent to the ward. Considering the pharmacokinetic/pharmacodynamic (PK/PD) variability and therapeutic index of cytotoxic drugs, they are used in a variety of ways to meet different clinical needs, such as outpatient transportations, implantable devices, venous pumping, intravenous infusion, intravenous injections, and ambulatory home chemotherapy (7, 8). The duration of the administered infusion solutions is often prolonged, even extending to several days; for example, ifosfamide may be administered as a continuous home-based infusion over 14 days for the treatment of soft tissue sarcoma (7). The physicochemical stability and sterility of cytotoxic drug-administered infusion solutions, which are crucial for ensuring their safety and effectiveness in clinical use, have been questioned (9).

The administered infusion solution check is a vital step in the PIVAS workflow and serves as a guarantee for drug safety. However, there is no associated report emphasizing and summarizing the aspects of quality control. A European conference consensus addressed the practical stability of post-dilution or post-reconstitution but did not define the technical term “administered infusion solution,” which has been used for several years in China (9, 10). There are many factors that affect the quality of administered infusion solutions, such as diluent, concentration, container, temperature, and duration of storage. Quality control mainly includes the assessment of physical stability (visual appearance, pH, osmolality, and particle formation), chemical stability (content), and sterility (microbiology and endotoxin). In this context, we collected the data related to cytotoxic drug-administered infusion solutions. This paper mainly evaluates the stability and sterility of cytotoxic drugs in administered infusion solutions. The findings provide an important reference to evaluate the quality control of administered infusion solutions.

## 2 Factors

Cytotoxic drugs commonly used in clinical practice were selected from the medical insurance catalog in China. Intravenous administration includes intravenous injection and intravenous infusion. This paper mainly focuses on cytotoxic drugs administered by intravenous infusion, and injectable drugs such as Bleomycin and azacitidine are excluded. The stability of a single drug in a single solvent was screened, while the stability of multi-drug mixtures was not within the scope of this study. The quality control of administered infusion solutions includes the physical stability, chemical stability, and biological stability. As there is no specific standard to investigate the quality of administered infusion solutions, we mainly refer to the criteria of injection stability in the 2020 Chinese Pharmacopoeia. These criteria mainly include assessments of visual appearance, particulate matter, pH, content, and sterility. All results are presented in Table 1.

**TABLE 1:** Status analysis of the cytotoxic drug-administered infusion solution quality control.

Drug	Factors					Physical stability				Chemical stability		Sterility	Package Insert	Ref
	Diluent	Container	Concentration	Temperature	Light	Visual appearance	pH	Osmolality (mOsm/L)	Particulate matter	limitation threshold	Content			
Cyclophos-phamide	NS 5%GS	PVC bag	0.3,2.0 mg/mL	4°C 23–25°C	light- exposure	no change	NA	NA	NA	10%	8d, 4°C 4d, 25°C	NA	<8°C, 24 h	(27)
	NS 5%GS	PVC bag; Glass bottle	1,20 mg/mL	4°C 23–25°C	light-prote-ction	NA	NA	NA	NA	10%	7d, 4°C 7d ,23−25°C	NA		(19)
Ifosfamide	NS 5%GS	PVC bag	30 mg/ml	4°C	light-protection	Clarify No color change No precipitation	NA	NA	NA	10%	30d, 4°C	NA	NA	(28)
Busulfan	NS	PVC bag	0.54 mg/ml	2-8°C 23–27°C	light-protection	No color change No precipitation	NA	NA	NA	10%	3h,25°C;30h, 2−8°C	NA	NS, 5%GS, 25°C, 8 h NS, 2–8°C 12 h	(29)
	NS	PVC bag; Glass bottle	0.55 mg/ml	2–8°C 20 ± 5°C	NA	random appearance of precipitation	−1.2pH unit, 6 h, in PVC bag; −0.5pH unit, 6h, in glass bottle	Change, 48 h, 20 ±5 °C	NA	5%	2−8°C 14 h, in glass bottles; 6 h in PVC bag	NA		(30)
Dacarbazine	5%GS	PVC bag; glass bottle	11 mg/ml in glass bottle; 1.4 mg/ml in PVC bag	4°C 25°C	light- exposure /light-protection	96h color change 24 h appearance of precipitation	±0.45pH unit	NA	NA	90-105%	glass bottle:24 h, 25°C in the light; 96 h,4°C in the dark PVC bag: 2 h, 25°C in daylight, 24 h in fluorescent light, 72 h in the dark;168 h, 4°C	NA	NA	(16)
Temozolomide													14 h, 25°C	
Carmustine	5%GS	PP bag; PE bag	0.2, 1.0 mg/mL	2–8°C 22°C	light-protection	No color change No precipitation	−1.3pH unit, 48h	No change	NA	10%	60 h 2-8°C 8.5h,22°C	NA	8 h, 25°C; 24 h, 2–8°C	(14)
	5%GS	glass bottle; PVC bag	0.1, 0.5 1 mg/mL	4°C 22°C	light-protection	NA	NA	NA	NA	10%	4°C, 48 h, in glass bottle,12 h, in PVC bag 25°C 2.5 h, in glass bottle 1 h, in PVC bag	NA		(31)
Bendamustine	NS	PO bag	0.25 0.60 mg/mL	2–8°C 23–25°C	NA	NA	NA	NA	NA	5%	3.5 h, 23–25°C 48 h, 2–8°C;	NA	24 h 2–8°C; 3 h, RT	(32)
Methotrexate	NS 5%GS	PO bag	0.2, 20 mg/mL	25°C	light-protection	Clarify; No color change No precipitation	NA	NA	NA	5%	NS, 28d,0.2, 20 mg/ml; 5%GS,28d, 20 mg/ml,3d, 0.2 mg/ml	NA	NA	(15)
Pemetrexed	NS	PO bag	2,13.5 mg/mL	2–8°C	light-protection	Clarify No color change No precipitation	±0.43Ph	NA	2mg/mL: ≤200 particles ≥10 μm per 100 mL, ≤50 particles ≥25 μm per 100 mL; 13.5 mg/mL: ≤550 particles ≥10 μm per 100 mL and ≤50 particles ≥25 μm per 100 mL	5%	28d 2–8°C	NA	24 h, <8°C	(33)
	NS 5%GS	PO bag	4, 9 and 12 mg/mL	2–8°C 22–25°C	light- exposure	browning on 2d, 25°C; 5d, 4°C No precipitation	NA	NA	NA	10%	4d, 2–8°C 1d, 22–25°C	NA		(17)
	NS 5%GS	PVC bag	2, 10, and 20 mg/mL	4°C 23°C	light-protection	Clarify No precipitation	NA	NA	large numbers of particulates at 24h	5%	31d, 4°C;2d, 23°C			(18)
Raltitrexed													24 h, <8°C	
Fluorouracil	NS	PO bag	7,8 mg/ml	23 ± 2°C	NA	Clarify No color change; no precipitation	8.79 ± 0.05 pH unit, no change	NA	NA	5%	7 mg/ml, 17d; 8 mg/ml, 24d	NA	4 h, 25°C	(25)
	NS 5%GS	PO bag	20,30, 40,50 mg/mL	2–8°C 25°C	NA	Clarify No color change;	9.15–9.26pHunit, no change	NA	NA	10%	15d, 2-8°C/25°C	NA		(34)
	NS	PVC bag	8 mg/mL	5°C ± 3°C	NA	Clarify No color change; No precipitation	8.82 ± 0.01pHunit No change	NA	NA	5%	28d, 5°C ± 3°C	NA		(35)
	NS 5%GS	PVC bag	1,10 mg/ml	4°C, 21°C	NA	Clarify No color change No precipitation	No change	NA	NA	10%	14d, 4/21°C	NA		(36)
Ftorafur														
Cytarabine	NS	glass bottle	1,5,10 mg/mL	2–8°C 25°C	light-protection	clarify no color change no precipitation	NA	NA	NA	5%	4°C,28d;25°C, 1 mg/mL, 14d,5 mg/ml,8d, 10 mg/mL, 5d	NA	24h, 4/25°C	(26)
Gemcitabine	NS, 5%GS	PVC bag	0.1,10 mg/mL	4, 23°C	light- exposure /light-protection	clarify, no color change no precipitation	NA	NA	NA	5%	35d, 4/23°C	NA	NS, 24 h, RT	(37)
Decitabine	NS	PO bag	0.334 mg/mL	2–8°C	light-protection	NA	NA	NA	NA	5%	48 h, 4°C	NA	4 h, 2–8°C	(38)
	NS	PO bag	0.5 mg/mL	2–8°C	light-protection	clarify, no color change no precipitation	6.9 pH No change	NA	NA	10%	24 h 2–8°C	NA		(39)
Fludarabine	NS	PE bag	0.05 mg/ml	2–8°C 15–25°C	light-protection	NA	NA	NA	NA	10%	15d, 2–8°C/15–25°C	NA	24 h, 4°C;8 h, 25°C	(3)
Daunorubicin	NS, 5%GS	PVC bag	100 μg/ml	4°C 25°C	light-protection	no color change no precipitation	No change	NA	NA	10%	43d, 4°C/25°C	NA	24 h, 25°C;48 h, 4°C	(40)
	NS, 5%GS	PVC bag	16 μg/ml	4°C	light-protection	NA	NA	NA	NA	10%	7d, 4°C	NA		(41)
Epirubicin	NS, 5%GS	PVC bag	100 μg/ml	4°C 25°C	light-protection	no color change no precipitation	No change	NA	NA	10%	NS, 20d, 25°C NS, 5%GS, 43d, 4°C	NA	NA	(42)
	NS, 5%GS	PVC bag	40 μg/ml	4°C	light-protection	NA	NA	NA	NA	10%	7d, 4°C	NA		(41)
Doxorubicin	NS, 5%GS	PVC bag	100 μg/ml	4°C; 25°C	light-protection	no color change no precipitation	No change	NA	NA	10%	NS, 23d, 25°C NS, 5%GS, 43d, 4°C	NA	NA	(40)
	NS, 5%GS	PVC bag	0μg/ml	4°C	light-protection	NA	NA	NA	NA	10%	7d, 4°C	NA		(41)
Pirarubicin	5%GS	PVC bag	800 μg/ml	4°C	light-protection	NA	NA	NA	NA	10%	5d, 4°C	NA	NA	(41)
Aclarubicin														
Etoposide	NS, 5%GS	PO bag	0.38, 0.74, 1.26 1.75 mg/mL	2–8°C 25°C	light-protection	white precipitate at 16d at 2–8°C in 5% GS at 1.75 mg/ mL; white precipitate	±0.15 pH unit	NA	NA	10%	61d, 25°C in 5%GS at 0.38, 0.74 ,1.26 mg/mL; 28d, 25°C in 5%GS at 1.75 mg/mL 16d, 25°C	NA	96 h at 0.2 mg/mL at 25°C, 48 h at 0.4 mg/mL at 25°C	(42)
	NS	Glass bottle, PVC bag, PE bag	0.4 mg/ mL	2-8°C 22-26°C	light- exposure	no color change, Precipitationat 48 h in glass bottle at 2–6°C, 72h in all the containers at 22–26°C	3.8_4.1	NA	NA	10%	24h, 4°C/25°C in glass or PE bag; unstable in PVC	NA		(43)
Homoharringtonine														
Teniposide														
Topotecan	NS, 5%GS	PVC bag	10, 25, 50 μg/ml	2-8°C 25°C	light-protection	no color change no precipitation	3.6–3.1pH unit, no change	NA	NA	5%	28d, 4/25°C	NA	24 h, <30°C	(44)
	NS, 5%GS	Glass bottle, PVC bag, PO bag	25, 50 μg/ml	5°C 23–24°C	NA	no color change no precipitation	3.31–3.58 pH unit, no change	NA	NA	5%	7d, 5°C 24 h,23–24°C	NA		(45)
Elemene														
Irinotecan	NS, 5%GS	PVC bag	0.4, 1.0, 2.8 mg/ml	2–8°C 25°C	light- exposure /light-protection	no color change no precipitation under light-protection color change, precipitation formation under light- exposure	4.2–3.6 pH unit, no change	NA	NA	10%	28d, 4/25°C under light-protection; 7d, 4/25°C under light- exposure	NA	6 h, 25°C; 24 h,2–8°C	(46)
Vindesine														
Vincristine	NS	PVC bag	10,20,40,60 μg/ml	4°C 23°C	light-protection	clarify, no color change no precipitation	NA	NA	≥ 10 µm, few	5%	9d, 23°C	NA	NA	(47)
Vinorelbine	NS, 5%GS	PVC bag	0.5, 2 mg/mL	25°C	light- exposure	clarify, no color change no precipitation	NA	NA	NA	10%	120h, 25°C	NA	24 h, 5–30°C	(48)
	NS, 5%GS	PVC bag	0.5 mg/ml	4°C	light-protection	NA	NA	NA	NA	10%	7d, 4°C	NA		(49)
Paclitaxel	NS, 5%GS	Glass bottle, PO bag, PE bag	0.3,1.2 mg/ml	2–8°C 25°C	light-protection	13d no color change, at 2-8°C 3d no color change at 25°C 0.3 mg/ml, 15d precipitation formation at 2-8°C 1.2 mg/ml, 10d precipitation formation at 2-8°C 0.3, 1.2 mg/ml,3d precipitation formation at 25°C	3.56 ± 0.5pH unit, no change	NA	≥ 10 µm/ml, at 3d exceed 25	5%	0.3 mg/ml in NS for 13d (PO bag, glass bottle), 16d(PE bag) at 2-8°C 0.3 mg/ml in 5%GS for 13d (PO bag), 18d(PE bag), 20d(glass bottle), at 2–8°C; 0.3 mg/ml, 3d, 25°C 1.2 mg/ml in NS for 9d (PO bag), 12d(PE bag), 10d (glass bottle) at 2-8°C; 1.2 mg/ml in 5%GS for 10d (PO bag, glass bottle), 12d(PE bag) at 2–8°C 1.2mg/ml, 3d, 25°C	NA	27 h, 25°C	(50)
Nedaplatin														
Cisplatin	NS	glass bottle, PE bags	0.1,1 mg/ml	15–25°C	light-protection	clarify, no color change no precipitation	no change	NA	NA	10%	30d, 15-25°C	NA	NA	(51)
Carboplatin	NS	PVC bag	0.5, 2.0, 4.0 mg/mL	4°C 25°C	at 25°C under light- exposure and 4°C under light-protection	clarify, no color change no precipitation	NA	NA	≥10 μm from 2 to 30 particles	10%	7d, 4°C 0.5 mg/ml,3d; 2.0 mg/ml,5d;4.0 mg/ml, 7d at 25°C	NA	8 h, 25°C 24 h, 4°C	(52)
	5%GS	PVC bag	0.5, 0.75, 2, 4 mg/mL	4°C 25°C	light-protection	clarify, no color change no precipitation	4.6–4.7, no change	NA	NA	10%	21d, 4°C/25°C	NA		(53)
Loplatin														
Oxaliplatin	5%GS	PVC bag, PP bag, PE bag	0.2,1.3 mg/ml	4°C 20°C	at 25°C under light- exposure and 4°C under light-protection	clarify, no color change no precipitation	4.5–4.7 pH unit, stable	NA	NA	10%	14d, 4°C/25°C	No microbial contamination	24 h, 2–8°C	(24)
	5%GS	PO bag	0.7 mg/ ml	3-7°C 20–24°C	at 25°C under light- exposure and 4°C under light-protection	clarify, no color change no precipitation	stable	NA	No particulate matter	10%	30d, 4°C/25°C	NA		(54)
Eribuline	NS	PE bag	20 μg/ml	2-8°C 25°C	light-protection	clarify, no color change no precipitation	stable	NA	NA	10%	28d,4°C/25°C	NA	4 h, 25°C;24 h, 4°C	(55)
	NS	PO bag	15.4, 43.3 μg/ml	4°C 20°C	at 25°C under light- exposure/ light-protection and 4°C under light-protection	clarify, no color change no precipitation	stable	NA	NA	10%	14d,4°C/25°C	NA		(56)

### 2.1 Diluent

For continuous venous infusion, the drug is diluted in a suitable injection solution. The pH value and electrolyte composition of the diluent are important considerations. Therefore, under normal circumstances, diluents with a pH similar to that of the drugs are usually selected for dissolution and dilution. The diluent listed in the drug instructions should be used, and non-electrolyte solvents should be selected for drugs sensitive to electrolytes (11). The commonly used diluents for infusion solutions include 5% and 10% dextrose (5% and 10% GS), 5% glucose sodium chloride, and 0.9% sodium chloride (NS). Most studies included in our analysis chose NS and 5% GS as solvents to evaluate the stability and quality of administered infusion solutions.

### 2.2 Container

The containers for infusion solutions are often made of glass or plastic, such as bottles, cassettes, syringes, or bags. Plastic containers offer several advantages, such as small size, lightweight, ease of storage and shipping, and being unbreakable. These advantages have led to plastic gradually replacing glass bottles as the main material for infusion solution containers. At present, the plastic bags used in the pharmaceutical industry, at home and abroad, are made of polyvinyl chloride (PVC), polyethylene (PE), polypropylene (PP), polyolefin (PO), and ethylene–vinyl acetate (EVA). PVC bags contain the plasticizer di-(2-ethylhexyl) phthalate (DEHP) to make them soft and flexible, but leaching of harmful substances like DEHP into the solutions has been reported (12). Carmustine has been reported to adsorb onto PVC and EVA surfaces but not onto PE or glass containers, making it suitable only for PVC-free containers like PE or PP bags (13, 14). So, new types of infusion bags made from materials such as PP and PE supersede the PVC as the main material. In our paper, we list the containers used for the quality control of administered infusion solutions. Many articles were published years ago on the use of PVC bags, so we include some recent studies in our research. However, the available literature in this area remains relatively scarce.

### 2.3 Concentration

Concentration is crucial for cytotoxic drugs as the clinically relevant dose intervals range from tens of milligrams to a several grams, and therefore, diluted solutions are prepared in a wide range of concentrations. Some cytotoxic drugs have different indications at different doses; for example, high doses of methotrexate are used for breast cancer and head and neck cancer, while low doses are used for autoimmune diseases. Different concentrations also affect the stability of the administered infusion solution. For example, high concentrations of methotrexate (20 mg/mL) can be stored at room temperature for 28 days, whereas low concentrations of methotrexate (0.2 mg/mL) can only be stored for 3 days under the same conditions (15). We list concentration as the one of the factors influencing the quality of cytotoxic drug-administered infusion solutions.

### 2.4 Temperature

Temperature is one of the main factors affecting the stability of administered infusion solutions. In clinical practice, two temperature conditions, namely, room temperature (RT, 22°C–25°C, 25°C ± 2°C, and 25°C) and refrigeration (2°C–8°C, 4°C ± 3°C, and 4°C), were used for storing ready-to-use solutions.

### 2.5 Light

Light is the most important factor influencing the stability of the drug. Sunlight can have a dramatic effect on the stability of diluted solutions in polypropylene containers. Light protection is important for many cytotoxic drugs; for example, methotrexate and dacarbazine should be protected from direct sunlight during storage (16). We considered the effect of light on the physicochemical stability of administered infusion solutions, and most articles reviewed in our research studied this aspect.

## 3 Cytotoxic drug-administered infusion solution stability

The stability of injection includes physical stability, chemical stability, and biological stability. There is no relevant standard for the stability of administered infusion solutions, so we chose the standard of injection stability from the 2020 Chinese Pharmacopoeia as a reference to evaluate their quality.

### 3.1 Physical stability

Physical stability generally refers to changes in the physical properties, mainly including visual appearance, pH, osmolality, and particulate matter (Table 1).

#### 3.1.1 Visual appearance

Visual appearance includes solution color, turbidity, and precipitation, and it is the first step in the quality control of administered infusion solutions. Pharmacists and nurses can directly observe the changes to ensure drug safety. The administered infusion solution shall be visually inspected under natural light, and there are no foreign bodies such as glass chips, fibers with length >2 mm, lumps with maximum particle size >2 mm, and unshaken particles. Although the chemical content is within limits, its color has changed, or some precipitation has formed in the long-term storage (17, 18). Therefore, visual appearance is important for the quality control of cytotoxic drug-administered infusion solutions (Table 1).

#### 3.1.2 pH

pH is a numerical indicator that describes the degree of pondus hydrogenii of a solution. pH is an important parameter for the stability of injections according to the 2020 Chinese Pharmacopoeia as changes in pH can affect the solubility of the drug and cause precipitation. The normal physiological pH of the human body is 7.35–7.45. Injections with extreme pH levels are more likely to cause vascular irritation, inflammatory reactions, or pain. Extreme pH (pH > 10 and <2) can increase the degradation rate of drugs such as cyclophosphamide (19). In order to increase drug availability and decrease the risk of local irritation, the pH limits are within 3.5–9 (20). The latest expert consensus in 2023 recommends that pH value variations less than 10% indicate that the administered infusion solution is stable (10, 21). We found that most articles did not provide enough detail about pH, such as terms like stable or unchanged, and some articles did not mention this parameter at all. We have listed these data according to the original data in the literature (Table 1).

#### 3.1.3 Osmolality

Osmolality means the extra pressure applied on the surface of the solution just enough to prevent osmosis from occurring. Osmolality is another important parameter that must be considered regarding the quality control of administered infusion solutions. The osmolality of the blood is 285–310 mOsmol/kg, and the average physiological osmolality is approximately 297.5 mOsmol/kg. Either hypertonic solutions with an osmolality >600 mOsmol/kg or hypotonic solutions with an osmolality approximately <150 mOsmol/kg have been reported to possibly cause crenation as an adverse reaction to intravenous infusion (22). Each drug has a different osmolarity; compound such as 0.9% NaCl and 5% dextrose, as isotonic solutions, are used as diluents for intravenous administration in clinical practice. The latest expert consensus in 2023 recommends that the osmolality changes of 10% indicate that the solution is stable and physically compatible compared with the initial condition (0 h) (10). Most research studies selected in this paper do not list exact osmolality or do not explore this factor (Table 1).

#### 3.1.4 Particulate matter

Particulate matter refers to small insoluble substances that are generally less than 50 μm and invisible to the naked eye. Infusion particles are those that enter the human body during the intravenous infusion process and cannot be removed from the body. The 2020 Chinese pharmacopoeia *stipulates that* particles ≥ 10 μm/mL should not exceed 25, particles *≥* 25 μm/mL should not exceed 3, and particles ≥50 μm should not be detected in more than 100 mL of an intravenous solution. The particles ≥ 10 μm should not exceed 6000, and particles ≥25 μm should not exceed 600 in each test sample for less than 100 mL of the intravenous solution. During infusion, the particles will adhere to the blood vessel wall and aggregate into larger particles, which may block microvessels, resulting in local blood vessel obstruction and insufficient blood supply. In addition, these particles will stimulate the body to produce allergic reaction and form granulomas. It is important to systematically evaluate the particulate matter. However, most studies focusing on the stability of ready-to-use infusion solutions do not list the data on particulate matter, highlighting the lack of standards and data on the administered infusion solutions (Table 1).

### 3.2 Chemical stability

Chemical stability refers to the content change due to hydrolysis or oxidation chemical degradation reactions (Table 1). The content of a drug will change over time, depending on the duration of storage and temperature. In clinical practice, given the general rule that drugs remain (i.e., at the recommended dilution) at up to 90% of their initial content, this 10% degradation has been widely used as the stability limit in published studies. Each drug has different pharmacodynamics, which results in different levels of stability. For anticancer drugs, which have a narrow therapeutic range for effective treatment, the classical 10% degradation limitation may not be appropriate. In such cases, the stability limitation is set at 5% or in compliance with the relevant standards in the 2020 Chinese Pharmacopoeia. However, most of the reference studies reviewed in our text chose 10% limitation, with only a few studies employing 5% or 95%–105% as the stability limit.

### 3.3 Biological stability

Biological stability generally refers to the deterioration and corruption due to microbial exposure. Sterility is another important factor in evaluating the stability of injections according to the 2020 Chinese Pharmacopoeia (Table 1). Administered infusion solutions are post-dilution or post-reconstitution products in PIVAS centers; however, there is no sterility data on administered infusion solutions. Most pharmaceutical industries frequently limit the use to 24 h after post-dilution or post-reconstitution only for bacteriological reasons, regardless of the true clinical practice, which, in many cases, could be longer. In practice, some cytotoxic drugs, such as 5-FU, may require continuous infusions for several days, so it is vital to test sterility (23). In this paper, we list sterility as an important part of administered infusion solution quality control. Only one article deals with microbial contamination of administered infusion solutions in our research (24). It demonstrated that the current attention to the quality control of administered infusion is not enough.

## 4 Discussion

The quality control of administered infusion solutions is a vital step from PIVAS to the ward and is indispensable to ensure the proper use of drugs. The quality control of cytotoxic drug-administered infusion solutions is important for patient safety in clinical practice and should be strengthened. In the paper, we summarized and listed the cytotoxic drug-administered infusion solution-associated data, mainly including the physical and chemical stability and sterility. However, there are some limitations to this analysis.

Many drugs selected are not commercially available products but are standard products for laboratory use only and, therefore, do not reflect clinical practice. This also means that pharmaceutical excipients in the commercial product cannot be analyzed, which prevents an accurate reflection of the drug’s true content in the administered infusion solution. In some cases, the admixtures were prepared in a laboratory to mimic a real-world environment in PIVAS centers; therefore, this information should be carefully applied for real-world practices.

The same drug may have multiple manufacturers, and the pharmaceutical excipients used by each manufacturer are different. Is it necessary to compare each product in the market? Can the analysis of only one product be used to represent all the other products in the market? In our research, we mainly listed one product for each drug. We also found that many drugs lack information regarding administered infusion solutions, especially some original products such as elemene and ifosfamide. Some frequently used cytotoxic drugs such as loplatin and nedaplatin also lack associated data on administered infusion solutions.

When reviewing package inserts, information regarding post-dilution or post-reconstitution is frequently limited to 24 h to meet licensing requirements. When medicines are being licensed, little attention is given to drug administration in the clinic. Therefore, pharmacists and medical staff need to pay more attention to the preservation and use of administered infusion solutions to ensure patient safety. It is vital to fill the gap between the package insert and clinical needs.

Most tertiary hospitals in China are equipped with PIVAS centers, where intravenous solutions are admixed. Although the environment in PIVAS centers is clean, it is necessary to check the sterility of administered infusion solutions after prolonged storage. Any microbial contamination can lead to serious effects in patients. There has been limited research on the sterility of administered infusion solutions. In addition to potential time-dependent physical and chemical changes, microorganism contamination poses a risk during post-dilution storage. The monitoring of heat sources and endotoxins after long-term storage of administered infusions is also crucial. Although most administered infusion solutions are used within 12 h of admixture, sterility should be the first consideration under some special conditions, such as continuous pumping for a long time, home infusion, and changing infusion time due to disease progression. Almost all tertiary hospitals can carry out microbiological examination, so pharmacists or nurses should take sterility into consideration to ensure drug safety for patients and avoid unnecessary waste.

With the continuous development of the tiered diagnosis and treatment model in China, daytime chemotherapy and home chemotherapy will become new models for the diagnosis and treatment of cancer patients. However, due to a lack of data on administered infusion solutions, the widespread application of these models to all chemotherapy regimens is limited. Improving these data will have huge impact on patients, the pharmaceutical industry, nursing staff, and economic aspects.

Another limitation to our research is the lack of associated data on degradation products, which is essential for the quality control of administered infusion solutions, especially for cytotoxic drugs. For example, high doses of 5-FU could increase cardiotoxicity because of small quantities of degradation products (fluoromalonaldehyde and fluoroacetaldehyde) produced during storage in administered infusion solutions (25). The limitations vary for each drug, and the content of by-products should be strictly detected, following the ChP criterion. Some drugs may retain more than 95% of their initial content; however, when degradation product levels exceed ChP limitations, it could compromise the chemical stability of infusion solutions due to the degradation product (26).

## 5 Conclusion

In order to standardize quality inspection, improve the quality control of the cytotoxic drug-administered infusion solutions, and ensure the safety and effectiveness of intravenous drug use, this paper established a quality control paradigm based on practical clinical needs including visual appearance (color, turbidity, and visual foreign body), pH, osmolality, particulate matter, drug content, and sterility. These indexes are analyzed comprehensively to assess the quality and stability of the administered infusion solution. At present, there is limited research on cytotoxic drug-administered infusion solutions, and there is a lack of relevant standards and technical guidance. With the development of regionalized PIVAS centers, the clinical demand for research in this area is increasing, which may drive further investigation. The administered infusion solution quality control is vital for patients, pharmacists, and nurses in clinical practice and has huge economical potential, so it is important to enhance relevant research in this field.
